# Preoperative embolization strategy for the combined resection of replaced right hepatic artery in pancreaticoduodenectomy: a small case series

**DOI:** 10.1186/s40792-022-01403-y

**Published:** 2022-03-22

**Authors:** Shintaro Takeuchi, Yoshiyasu Ambo, Yoshihisa Kodama, Minoru Takada, Kentaro Kato, Fumitaka Nakamura, Satoshi Hirano

**Affiliations:** 1 Department of Surgery, Teine Keijinkai Hospital, 1-12-1-40, Maeda, Teine-ku, Sapporo, Hokkaido 006-8555 Japan; 2 Department of Radiology, Teine Keijinkai Hospital, 1-12-1-40, Maeda, Teine-ku, Sapporo, Hokkaido 006-8555 Japan; 3 Department of Gastroenterological Surgery II, Faculty of Medicine, Hokkaido University, West-7, North-15, Kita-ku, Sapporo, Hokkaido 060-8638 Japan

**Keywords:** Replaced right hepatic artery, Pancreaticoduodenectomy, Trans-arterial catheter embolization

## Abstract

**Background:**

Replaced right hepatic artery (rRHA) is a common vascular variation, and combined resection of this vessel is sometimes needed for the curative resection of pancreatic head malignancy. Safe surgical management has not been established, and there is a small number of reported cases. Here, we reported five cases, wherein preoperative embolization of rRHA was performed for combined resection.

**Case presentation:**

All patients had pancreatic head malignancies that were in contact with rRHA. We performed a preoperative embolization of the rRHA before the scheduled pancreaticoduodenectomy for the combined resection. Arterial embolization was safely accomplished, and the communicating arcade from the left hepatic artery via the hilar plate was clearly revealed in all cases. Four patients underwent the operative procedure, except for one patient who had liver metastasis at laparotomy. No patient suffered from a severe abnormal liver function during the management; however, one patient had multiple liver infarctions during the postoperative course.

**Conclusions:**

Preoperative embolization for the combined resection of rRHA in pancreaticoduodenectomy can be a management option for the precise evaluation of hemodynamics after sacrificing rRHA. In our cases, arterial flow to the right liver lobe was supplied by the left hepatic artery via the bypass route, including the communicating arcade of the hilar plate.

## Background

Hepato–biliary–pancreatic surgeons frequently encounter the anatomical variation of the hepatic artery, with replaced right hepatic artery (rRHA) being one of the most common variations [1–4]. When a pancreaticoduodenectomy is performed for the malignancy of the pancreatic head, rRHA could be contiguous to the tumor, because it often runs into the pancreatic head or parenchyma [5]. Although pancreaticoduodenectomy (PD) combined with rRHA resection is often needed for complete microscopic margin clearance of the malignancy in the pancreatic head, this procedure carries the risk of liver ischemia, which causes liver infarction, abscess, and anastomotic failure of the cholangiojejunostomy [6]. The reconstruction of the hepatic artery is an efficient approach, whereas this procedure requires an advanced technique with microscopic surgery.

As an alternative approach, we have adapted preoperative embolization management for combined resection of rRHA in PD. This approach may enable preoperative evaluation, development of collateral vessels, and simulation of the hemodynamics of the liver. Recently, a French study treated 18 cases with this approach and reported its safety and feasibility [7]. In the present case series, we presented five cases of pancreatic head malignancy, who underwent a preoperative embolization of rRHA, and combined resection of rRHA with radical PD. In all cases, collateral arteries that ran into the hilar plate were seen on angiography immediately after embolization. These appearances are unique and significant for the management of PD, because hemodynamics after rRHA resection has not been clearly understood. We provided a summary of the short-term outcomes of these five cases, including the evaluation of collateral vessels by angiography.

## Case presentation

### Treatment strategy

We have adapted the planned preoperative embolization of rRHA in five patients who underwent PD with combined resection of rRHA in our institute between 2013 and 2019. The presence of tumor abutment or involvement of the rRHA on preoperative computed tomography (CT) was the indication for combined resection of the rRHA. The characteristics of the patients are summarized in Table 1.

**Table 1:** Patient characteristics of preoperative embolization of rRHA

Case	Age	Sex	Diagnosis	Preope Tx	NCCN^a^	UICC^b^	Procedure	rRHA resection	rRHA origin	rRHA-tumor
1	65	M	PDAC	No	R	cIII	SSPPD	+	CA	Abutment
2	65	M	PDAC	GS	BR	ycIII	Exploratory laparotomy	−	SMA	Involvement
3	75	F	DBDC	No	–	cIIB	SSPPD	+	SMA	Abutment
4	71	M	PDAC	No	R	cIIA	SSPPD	+	SMA	Abutment
5	67	F	PDAC	S-1 + RT GnP	LA	ycIIB	SSPPD	+	SMA	Abutment

*rRHA* replaced right hepatic artery; *PDAC* pancreatic ductal adenocarcinoma; *DBDC* distal bile duct cancer; *PreopeTx* preoperative therapy; *GS* gemcitabine + S-1; *RT* radiation; *GnP* gemecitabine + nab-paclitaxel; *R* resectable; *BR* borderline resectable; *LA* locally advanced; *SSPPD* subtotal stomach-preserving pancreaticoduodenectomy; *CA* celiac artery; *SMA* superior mesenteric artery

^a^NCCN: Resectability criteria from the NCCN Guideline version1.2021 Pancreatic Adenocarcinoma before surgery

^b^UICC: The UICC TNM classification 7th edition

Four patients were diagnosed with pancreatic ductal adenocarcinoma (PDAC), and the fifth was diagnosed with distal bile duct cancer. The PDAC lesion was resectable in two patients, borderline resectable in one patient, and one patient had locally advanced PDAC, according to the NCCN guidelines. The patient with borderline resectable PDAC (Case 2) presented with a tumor adjacent to the common hepatic artery and received neoadjuvant chemotherapy with gemcitabine and S-1. The patient with the locally advanced-type lesion (Case 3) presented with a tumor adjacent to the aorta and received multidisciplinary treatments, including chemoradiotherapy with S-1, followed by systemic chemotherapy with gemcitabine and nab-paclitaxel. The main tumor shrank partially after the therapies without developing distant metastasis; therefore, we decided to perform conversion surgery.

In all five cases, preoperative arterial embolization was safely accomplished by interventional radiologists using the coil or vascular plug, and the collateral artery was evaluated after embolization (4–15 days before surgery). Postponing the surgery was an option if complications such as severe liver dysfunction or infarction occurred after embolization. The arterial flow was reassessed using CT before surgery. We routinely performed follow-up CT scans on post-operative days 7–10 to identify occult liver ischemia, intra-abdominal abscess, and obstruction of vessels. We performed a subtotal stomach-preserving pancreaticoduodenectomy (SSPPD) in the four cases, but did not adapt the procedure for a case because of liver metastasis on laparotomy (Case2). In all four cases of SSPPD, we resected the rRHA with the main tumors without any reconstruction.

### A representative case

**Fig. 1:**
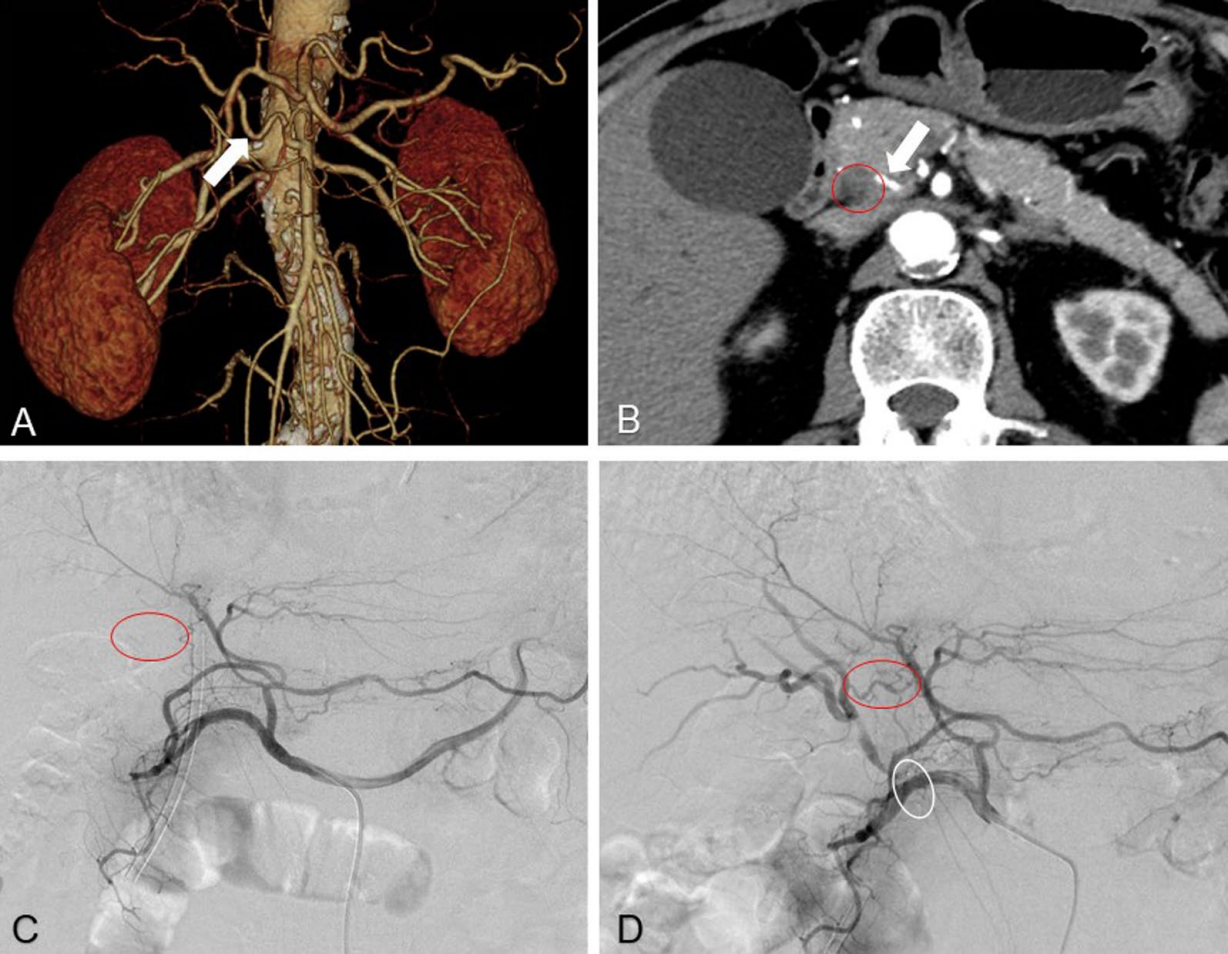
Case 4: Imaging findings of a representative case of preoperative embolization of rRHA. **A** rRHA arising from SMA by CT angiography (white arrow). **B** CT scan showed the main tumor of pancreatic head cancer (red circle) had closely contact with rRHA, which ran into the parenchyma of the pancreas (white arrow). **C** Before embolization of the rRHA, the vascular supply to the right lobe from the LHA was scanty (red circle). **D** After embolization of the rRHA by the plug (white circle), blood flow of right lobe of the liver was supplied via the communicating arcade from LHA, which went through the hilar plate (red circle). *rRHA* replaced right hepatic artery; *SMA* superior mesenteric artery; *CT* computed tomography; *LHA* left hepatic artery

We present a case representative of our management (Case 4 in Table 1). A 71-year-old man with pancreatic head cancer presented with an rRHA arising from the superior mesenteric artery that ran into the pancreatic parenchyma (Fig. 1A). Preoperative CT showed that the main tumor lesion had close contact with the rRHA (Fig. 1B). The patient was diagnosed with PDAC (stage IIA) based on a series of clinical examinations. Embolization was performed 4 days before the surgery. Arterial flow in the right lobe was not detected by selective angiography of the common hepatic artery (Fig. 1C) before embolization. Soon after the embolization, collateral arteries that ran into the hilar plate were seen on angiography (Fig. 1D). The patient did not have abnormal liver function, such as elevated serum liver enzyme or bilirubin levels after embolization. We performed SSPPD, wherein rRHA was dissected with the tumor. There were no complications related to liver ischemia, and the tumor was radically resected by pathology.

### Angiography

**Fig. 2:**
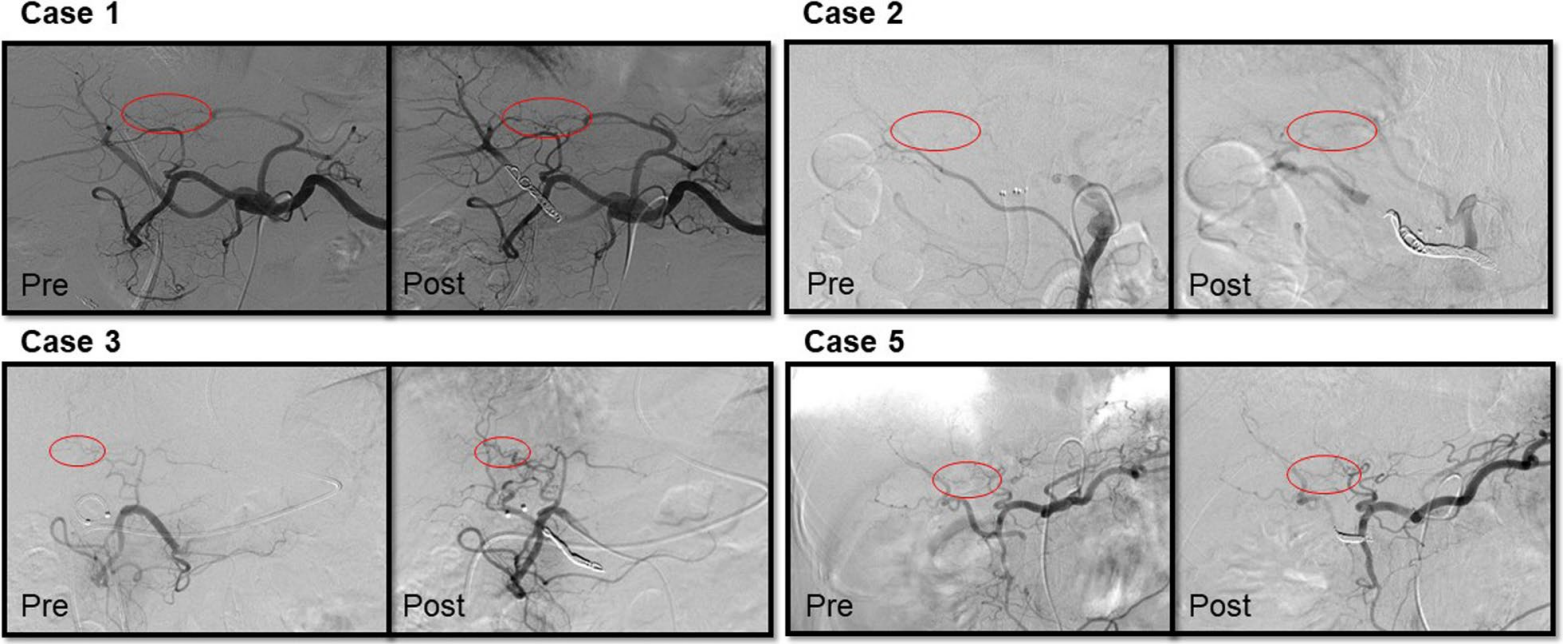
Angiography after the embolization of rRHA in the four cases other than case 4. In all cases, the blood flow to the right lobe of the liver was commonly supplied by the communicating arcade from the LHA via the hilar plate (red circle). These vessels were enhanced (Cases 1, 2, and 3) or retained (Case 5) after embolization. *rRHA* replaced right hepatic artery; *LHA* left hepatic artery; *Pre* pre-embolization of rRHA; *Post* post-embolization of rRHA

It is important to understand the dynamics of blood flow to the liver after resecting rRHA. Angiographic changes were observed in all five cases after rRHA embolization (Figs. 1D, 2). Confirmative angiography after embolization showed that the right hepatic artery was supplied by collateral blood flow from the left hepatic artery in all cases. This collateral route communicates to the arcade through the hilar plate, which is an important collateral blood flow connecting the left and right hepatic arteries [8]. This bypass route was retained or enhanced after embolization compared to that before embolization (Figs. 1D, 2).

### Short-term outcome

**Fig. 3:**
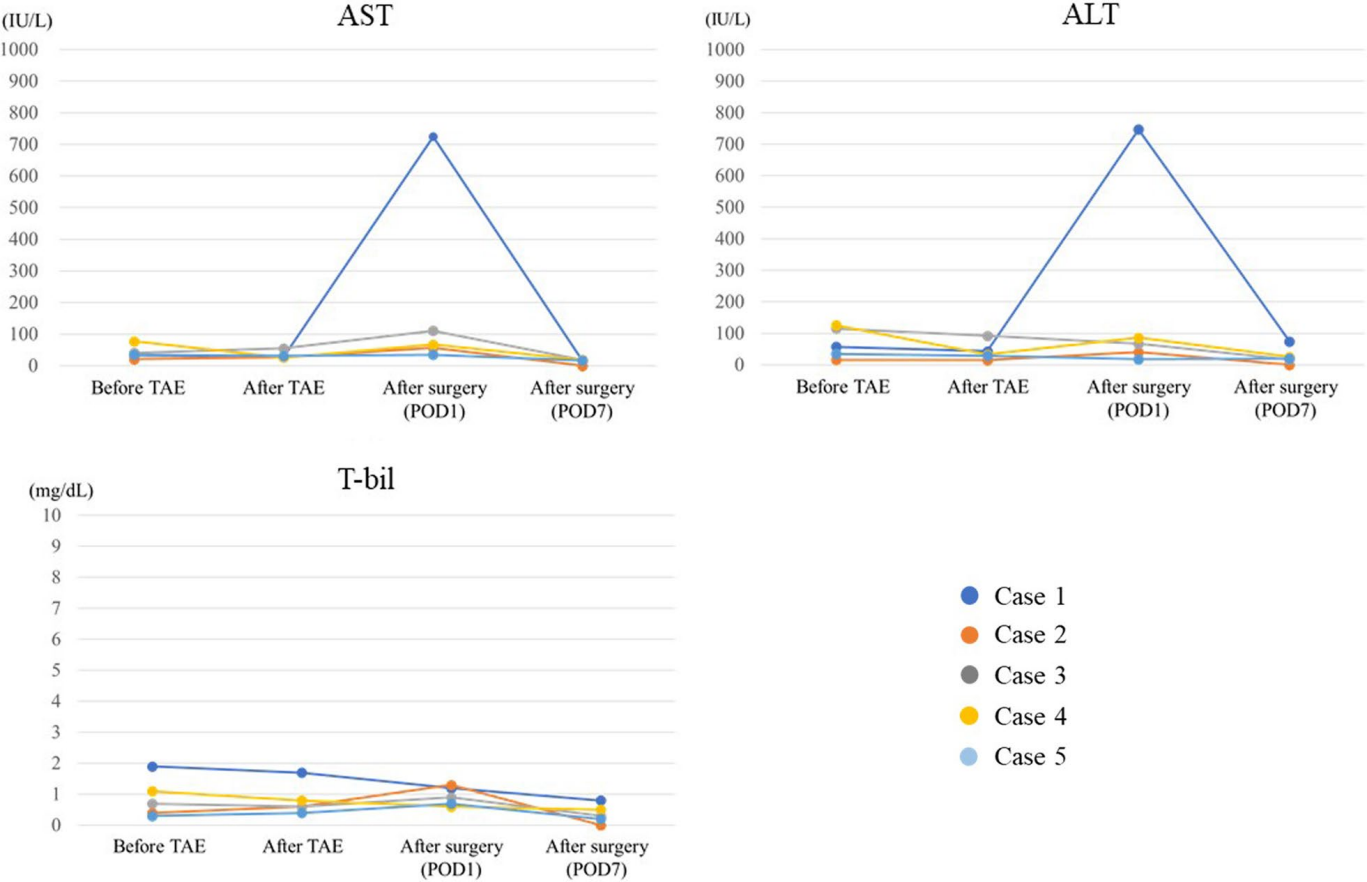
Laboratory data related to the liver function changing before or after the TAE or surgery. The elevation of AST or ALT was not shown in the clinical course other than their elevation at the postoperative day 1 of case 1. There was no elevation of T-bil in all cases during the processes. *TAE* trans-arterial catheter embolization; *AST* aspartate aminotransferase; *ALT* alanine aminotransferase; *T-bil* total bilirubin; *POD* postoperative day

The changes in laboratory data related to the liver function after embolization or surgery are shown in Fig. 3. No patients had an elevated serum liver enzyme or bilirubin levels after embolization. After surgery, although one case had a mild elevation of liver enzymes, the other four cases had a normal liver function. The clinical outcomes, including liver function or events associated with liver ischemia brought about by embolization or surgery, are summarized in Table 2. With regard to postoperative outcomes, four patients had no complications related to liver ischemia; however, there was a case of biliary tract cancer with multiple liver infarctions of the anterior and lateral segments without any laboratory changes in liver function (Case 3 in Table 2). The patient suffered from a cerebral infarction during the postoperative course. The patient was administered heparin as an anticoagulation therapy. Moreover, the patient had a hemorrhagic shock due to the bleeding from the arterial branch of the right femoral area, thus the need for an arterial embolization. Follow-up CT revealed multiple liver infarctions during these events (postoperative day 31), whereas the hepatic arterial flow was preserved on enhanced CT. We encountered a case of cholangitis (Case 5). The patient was diagnosed with cholangitis caused by enterobiliary reflux from the cholangiojejunostomy, as CT revealed that the arterial flow into the liver was preserved without ischemic change or abscess formation in the liver. The patient recovered within a few days after administration of antibiotics. All four resectable cases were pathologically diagnosed as R0 resection, defined as the absence of tumor cells along the resection margin. Preoperative and postoperative follow-up CT revealed that the arterial flow was maintained in the right hepatic lobe in all cases. There was no incidence of mortality within 90 days after surgery.

**Table 2:** Outcome of the patients

Case	Operative time (min)	Blood loss (mL)	Liver dysfunction		Complication	Clavien–Dindo classification^a^	R status of pathology
			After TAE	After surgery			
1	512	450	−	+	Pancreatic fistula	IIIa	R0
2	195	30	−	−	None	–	–
3	470	335	−	−	Pancreatic fistula	IIIa	R0
					Intraabdominal abscess	IIIa	
					Liver infarction	II	
					Cerebral infarction	II	
					Hemorrhage of right femoral area	IVa	
4	417	227	−	−	Intraabdominal abscess	IIIa	R0
5	572	610	−	−	Cholangitis	II	R0

*TAE* trans-arterial catheter embolization

^a^Dindo D, Demartines N, Clavien PA. Classification of surgical complications: a new proposal with evaluation in a cohort of 6336 patients and results of a survey**.** Ann Surg 2004;240:205–213

## Discussion

This report involved five surgical cases who underwent PD with combined resection of rRHA. All patients had a malignant tumor in the pancreatic head adjacent to the rRHA. We have adapted the surgical strategy, including preoperative embolization for rRHA, for these cases. In all cases, angiography showed an emergence of the communicating arcade from the left hepatic artery via the hilar plate soon after embolization. There was no liver dysfunction after embolization, so we could perform the surgical procedure except in the case of liver metastasis. A few previous studies have reported planned embolization of the rRHA for combined resection before PD performed with curative intent [7, 9, 10]. Our consecutive case series provided detailed clinical findings, including angiographic imaging and laboratory data, in these rare settings.

An rRHA arising from the superior mesenteric artery or common hepatic artery is the most common hepatic artery variation, which varies from 8 to 14% [3, 4, 11, 12]. This vessel is often encountered during PD, and the surgeon has to recognize its existence, as it courses under the pancreatic head or within the pancreatic parenchyma [5]. A study showed that the division of rRHA should be considered if tumors were situated within 10 mm from the root of this vessel to improve the rate of R0 resection [13]. For this reason, we performed PD with planned combined resection of rRHA for pancreatic head malignancies that had contact with this artery.

The standard treatment strategy for PDAC is undergoing change. Numerous studies have revealed the multimodal approach, including chemo(radio)therapy, ensures effective management of this disease [14, 15]. The oncological benefit of neoadjuvant treatment has been reported even in resectable cases [15, 16]. In general, the pathological margin status has been recognized as an important prognostic factor for malignancy of the pancreatic head [17, 18], and the effect of chemotherapy on the tumor margin can be evaluated only after resection. The opportunity for combined resection of the rRHA will not decrease even in the era of neoadjuvant treatment owing to the anatomical characteristics of the rRHA.

Ischemic changes in the liver or bile duct after the resection of the hepatic arteries can cause postoperative complications, such as hepatic infarction, hepatic abscess, or anastomotic failure of cholangiojejunostomy [6, 19–21]. The reconstruction of rRHA may be an effective approach to maintain blood flow in this artery [22, 23]. However, these procedures require a complex technique with microsurgery, and there may be a risk of severe hemorrhage from anastomotic breakdown if a pancreatic fistula occurs. Several studies or case reports have shown that preoperative embolization of the hepatic artery may lead to the development of collaterals from other arteries [7, 9, 24–26]. In all cases in the present report, the collateral artery from the left hepatic artery via the hilar plate was clearly demonstrated by angiography after embolization of the rRHA without any complications. As described in a recent study [7], this management approach could effectively protect the liver from ischemic changes.

After injury or occlusion of the hepatic artery, some intra- or extra-collateral arteries protect the liver from ischemia [27–29]. Previous studies have reported the communicating arcade via the hilar plate as the key vessel after occlusion or surgical resection of the opposite hepatic artery [8, 30]. We assessed the communicating arcade in all five cases. This collateral developed soon after embolization of the rRHA. There are some extra-hepatic collateral vessels in the liver, including the right inferior phrenic artery [31]. The blood supply from these arteries may prevent liver ischemia after rRHA resection without embolization. However, it is not clear which routes could function as major routes, because we did not conduct routine assessments of the extra-hepatic collaterals. Although a more comprehensive evaluation of extra-hepatic arteries which might work as a collateral route after embolization of rRHA is needed, we found that blood flow from the left hepatic artery via the communicating arcade in the hilar plate was one of the key routes in our setting.

In the present study, arterial embolization conducted by interventional radiologists was successful in all patients. However, major or minor complications associated with arterial embolization cannot be ignored [32], and proper placement of the coil or plug is needed for safe ligation of the rRHA during PD. Furthermore, a previous study showed that simple resection of the rRHA without preoperative embolization or reconstruction may be safe and feasible without severe morbidity [33]. Our study supports this concept, because all the patients demonstrated collateral arteries arising from the left hepatic artery without any occurrence of severe liver dysfunction after embolization or resection of the rRHA. As the collaterals may come into function soon after the ligation of the rRHA during surgery, as demonstrated by the results of angiography, preoperative embolization may be unnecessary. However, this previous study also reported a patient with insufficient collateral flow to the liver who developed a liver abscess [33]. This means that not all patients develop sufficient collateral arteries following rRHA occlusion. Therefore, our strategy, including preoperative rRHA embolization, could be useful for identifying such patients before surgery.

We encountered a case of multiple liver infarctions without liver dysfunction (Case 3). The patient had severe changes in vital signs due to hemorrhagic shock and other complications during the postoperative course. Total blood flow to the liver may decrease after resecting rRHA, and systemic blood flow reduction due to shock may cause liver ischemia. Marichez et al. [7] reported mortality in a patient who suffered from wide liver ischemia and severe sepsis without arterial obstruction. Thus, the rRHA embolization strategy may not ensure the safety of the liver. Careful management that prevents liver damage, such as proper fluid management, minimizing intraoperative bleeding, paying attention to preexisting atherosclerotic stenosis of the main arteries, and minimizing venous clamping time during portal vein reconstruction is essential even if the blood flow of the hepatic artery is preserved [7, 34].

The limitation of this report is that it was a small case series without a comparison between the cases. Although more cases are needed to verify the effectiveness or safety of our strategy, it could be a choice of surgical management for the malignancy of the pancreatic head with rRHA. Angiography findings after rRHA embolization could be useful for understanding hemodynamics after sacrificing this artery.

## Conclusions

From our five cases, preoperative embolization strategy for the combined resection of rRHA in pancreaticoduodenectomy could be a feasible choice for surgical management. The communicating arcade via the hilar plate from the left hepatic artery was one of the major routes bypassing the right hepatic lobe in these settings.

## Data Availability

Not applicable.

## Abbreviations

rRHA: Replaced right hepatic artery
PD: Pancreaticoduodenectomy
CT: Computed tomography
PDAC: Pancreatic ductal adenocarcinoma
SSPPD: Subtotal stomach-preserving pancreaticoduodenectomy
